# A Case of Isolated Splenic Tuberculosis Presenting as a Splenic Abscess Complicated by Rupture Leading to Splenectomy in an Immunocompetent Male

**DOI:** 10.7759/cureus.61088

**Published:** 2024-05-26

**Authors:** Rahul Ranjan, Ankita Rai, Jayanthi Gunasekaran, Talari R Paul, Rajiv M Gupta

**Affiliations:** 1 Department of Microbiology, Employees' State Insurance Corporation (ESIC) Medical College and Hospital, Faridabad, IND

**Keywords:** trace, cbnaat ultra, mycobacterium tuberculosis, splenectomy, chronic pancreatitis, pseudocyst, pyoperitoneum, splenic rupture, splenic abscess, spleen

## Abstract

We report a rare case of splenic tuberculosis (TB) in a male patient with a competent immune system who had no previous record of pulmonary TB. A 56-year-old male patient came to our outpatient department complaining of upper abdominal pain with a few episodes of vomiting for three days. He had alcoholism, smoked for 15 years, and had no past history of diabetes mellitus, hypertension, TB, or HIV. An abdominal ultrasound and CT scan at admission showed pancreatitis with a splenic abscess. After five days of admission, the patient's vitals deteriorated, and he had severe abdominal pain. CT scan suggested a splenic abscess rupture with hemoperitoneum. An emergency exploratory laparotomy was performed, and a splenectomy was done due to the splenic abscess rupture. A cartridge-based nucleic acid amplification test from splenic intracapsular fluid detected a trace *Mycobacterium tuberculosis* complex. The patient was discharged after starting first-line antitubercular treatment for six months. After three months of follow-up, the patient was doing well with no complaints.

## Introduction

Tuberculosis (TB) is a serious health issue in developing nations and remains one of the most prevalent and deadly infectious diseases. It affects millions of people worldwide, causing significant morbidity and mortality [1]. In 2022, TB held the unfortunate distinction of being the world’s second leading cause of death due to a single infectious agent, following closely behind the COVID-19 pandemic [2]. The reported global number of people newly diagnosed with TB reached a staggering 7.5 million in 2022. This figure represents the highest count since the World Health Organization initiated global TB monitoring in 1995 [2]. In cases of miliary or disseminated TB, the spleen emerges as the third most commonly affected organ, following the lungs and liver [3]. Splenic TB symptoms often lack specificity and can be misleading. Common manifestations include fever, abdominal pain, and weight loss, and usually, splenic TB becomes symptomatic when there is an abscess formation [4]. The case report outlines the clinical presentation, diagnosis, and treatment of splenic TB.

## Case presentation

A male patient, aged 56, came to our outpatient department, reporting upper abdominal pain for the past three days. He was in good health until this discomfort started. The pain was insidious in onset and gradually progressive. The patient also had a few episodes of vomiting, which mainly contained previously ingested food and was non-bilious.

The patient had been chronically consuming alcohol for 15 years, with a daily intake of about 375 mL of alcohol. He also had a smoking habit, consuming one to two cigarettes per day, and used to chew tobacco. However, he had ceased these habits for the past three months. The patient had no known history of chronic conditions such as diabetes mellitus or hypertension and no known allergies. There was no record of intravenous drug abuse or any other chronic illnesses. The patient's surgical history was unremarkable. He has not had TB in the past. Additionally, the patient's family history contained no significant health issues.

An ultrasound of the whole abdomen done two months before the presenting complaint demonstrated hepatomegaly with mild ascites with evidence of subacute intestinal obstruction.

**Figure 1:**
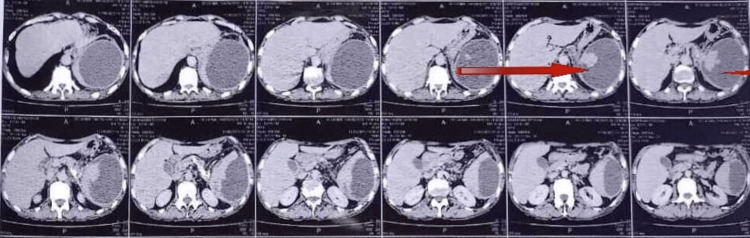
CECT of the abdomen axial section showing an atrophic pancreas and splenic abscess CECT - contrast-enhanced computed tomography

On admission, a contrast-enhanced computed tomography scan (CECT) of the abdomen (Figure 1 showing axial section) showed that the pancreas was atrophic in size with evidence of multiple tiny calcific foci along the duct and a dilated main pancreatic duct suggestive of changes of chronic calcific pancreatitis. The spleen could not be visualized with the presence of a large hypodense peripherally enhancing lesion in relation to the left hypochondriac region measuring approximately 12 × 10 cm. A loculated collection measuring approx. 8.7 × 5.5 cm was noted in the vesico-rectal space of the pelvic region. The liver was normal in size but showed reduced attenuation suggestive of fatty changes. Given these findings, the possibility of splenic hematoma or abscess formation could not be ruled out.

Upon examination, the patient's abdomen was found to be soft and not distended. Tenderness was observed in the left hypochondriac region and the epigastric area. A noticeable lump was also detected in the left hypochondriac region. There was no lymph node enlargement. The patient's pulse was recorded at 91 beats per minute, his respiratory rate was 16 per minute, and his blood pressure measured 132/82 mmHg. Based on these observations, the patient was admitted to our hospital with a preliminary diagnosis of chronic pancreatitis accompanied by a splenic abscess. Injection of ceftriaxone 1 gram IV BD, injection of metronidazole 500 mg TDS, and amikacin 500 mg IV BD were started empirically.

The patient's hemoglobin level was recorded at 7.7 g/dL, and the total leucocyte count was 8190 per microliter. The serum amylase and lipase levels were 423 IU/L and 257 IU/L, respectively (Table 1). The patient tested negative for HIV, HBsAg, and hepatitis C.

**Table 1:** Details the list of day-wise investigations conducted after admission CRP - C-reactive protein

Day of admission	Location	Random blood sugar (mg/dL)	Haemoglobin (g/dL)	Total leucocyte count (cells/uL)	Neutrophils (%)	Lymphocytes (%)	Monocytes (%)	CRP (mg/dL)	Serum amylase (IU/L)	Serum lipase (IU/mL)
Day 1	Ward	111	7.70	8190	62.2	19.7	14.2	-	423	257
Day 2	Ward	117	8.2	7330	57.4	22.1	15.7	-	-	-
Day 4	Ward	93	-	-	-	-	-	2.4	359	656
Day 6	A splenectomy was done and shifted to ICU (day 1)	-	11.3	6500	90.3	3.5	6.2	-	-	-
Day 7	ICU (day 2)	152	11.9	11280	89.4	2.8	6.3	-	-	-
Day 8	ICU (day 3), shifted to ward	130	10.0	17570	80	10	9.8	-	-	-

On day 2, a provisional diagnosis of splenic abscess with chronic pancreatitis with pseudocyst of the pancreas was made, and the patient was advised for exploratory laparotomy for further management, but the patient denied a major surgery so he was kept under observation and managed conservatively.

On day 5, the patient's condition deteriorated, and an ultrasound suggested a splenic abscess rupture. On day 6, an emergency exploratory laparotomy was performed, and a splenectomy was done due to the splenic abscess rupture. Splenic intracapsular fluid was sent for microbiological investigations. The splenic capsule and resected spleen were sent for histopathology. The patient was shifted to the ICU.

The Ziehl-Neelsen (ZN) stain was negative for acid-fast bacilli; however, the cartridge-based nucleic acid amplification test (CBNAAT) detected trace *Mycobacterium tuberculosis* complex and rifampicin resistance was indeterminate. The histopathology report showed evidence of splenic rupture with chronic inflammatory cells and infected hematoma.

On day 7, the chest X-ray showed an elevated left hemidiaphragm with radio-opacity in the left lower zone, causing the loss of normal cardiac silhouette, possibly representing partial atelectasis and consolidation of the left lower lobe, so to rule out pulmonary tuberculosis CBNAAT sputum was done and *Mycobacterium tuberculosis* complex (MTBC) was not detected. The postero-anterior (PA) view is shown in Figure 3.

**Figure 2:**
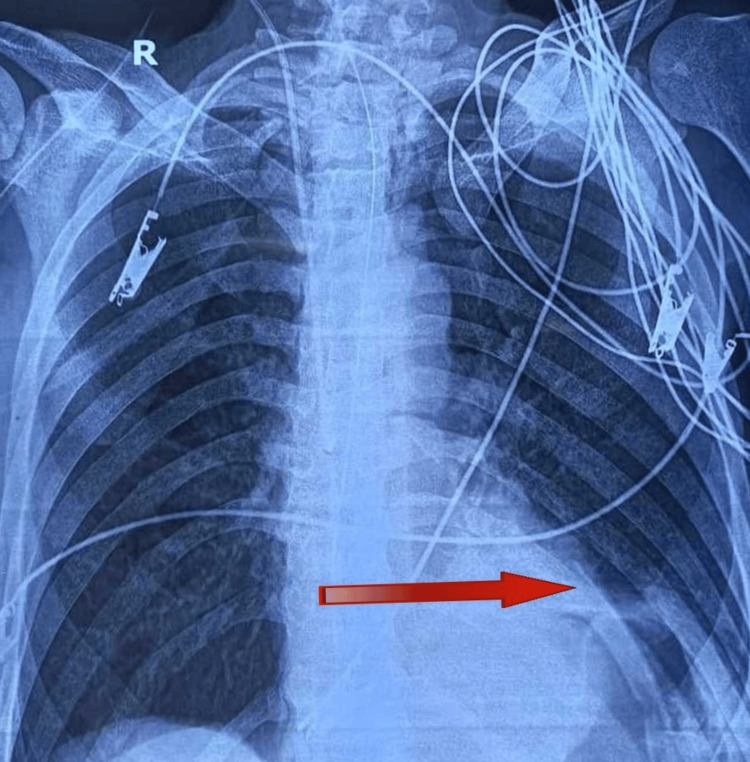
Chest X-ray postero-anterior view Chest X-ray showing an elevated left hemidiaphragm with radio-opacity in the left lower zone causing loss of normal cardiac silhouette, possibly representing partial atelectatic/ consolidation of the left lower lobe

On day 12, the patient was shifted to oral medications. Tab ceftazidime 500 mg twice daily and tab metronidazole 400 mg three times a day were started. On day 19 of admission, the patient was discharged after starting antitubercular treatment. In the intensive phase, four drugs were given orally, specifically rifampicin (8-12 mg/kg/day), isoniazid (4-6 mg/kg/day), ethambutol (12-18 mg/kg/day), and pyrazinamide (20-30 mg/kg/day). The patient proceeded to the continuation phase for four months with the following drugs for the completion of treatment: rifampicin (8-12 mg/kg/day), isoniazid (4-6 mg/kg/day), and ethambutol (12-18 mg/kg/day). On three separate three-month follow-ups, the patient was doing well with no new complaints.

## Discussion

TB is a significant infectious disease that remains prevalent in resource-poor countries. While its incidence has decreased in many developed nations, it continues to pose a significant public health challenge in many developing countries [5]. In 2022, TB tragically claimed the lives of approximately 1.3 million people worldwide. This devastating toll underscores the urgent need for continued efforts in prevention, diagnosis, and treatment to combat this infectious disease and save lives [2].

TB predominantly affects the lungs, accounting for 90% of cases. Splenic TB, which is an uncommon type of extrapulmonary TB, typically occurs as a secondary complication in miliary TB [1]. Splenic TB tends to occur more frequently in men [4]. Factors that increase the risk for splenic TB include immunodeficiency, infection with HIV, diabetes mellitus, abnormalities in the blood, long-term steroid therapy, and organ transplantation [1]. An Indian study on splenic TB found that 62% of the cases were linked with pulmonary TB, and half of the patients were coinfected with HIV [6]. In immunocompetent patients, the spleen can become infected through trauma, as well as through hematogenous spread from the lungs in cases of miliary TB and from nearby organs [5].

Splenic TB can be classified into five pathomorphological types: miliary, nodular, spleen abscess, calcific, and mixed type. Among these, the spleen abscess is the most common and typically presents with the most symptoms [7]. The clinical presentation of splenic TB can often be vague and nonspecific, which frequently leads to delayed diagnosis. The symptoms can vary from abdominal pain accompanied by an enlarged spleen to unexplained fever, paleness, fatigue, and weight loss. Splenic TB can also lead to serious complications, including hypersplenism, portal hypertension, and even rupture of the spleen [3,4].

Ultrasonography (USG) is often the first imaging technique used due to its cost-effectiveness and ability to help define the nature of the lesion. Lesions in the spleen on USG usually appear as several hypoechoic lesions, which could potentially be tuberculomas or abscesses [2]. The presence of organ involvement typically confirms a diagnosis of splenic abscess; thus, invasive diagnostic procedures are not commonly included as part of the routine. The diagnosis of isolated splenic TB is typically established through the pathological analysis of a fine needle aspiration biopsy, splenic biopsy, or specimen obtained from a splenectomy. In the meantime, the evaluation of adjacent organs, determination of the necessity for surgical intervention, and monitoring of treatment response are conducted using ultrasonography and computed tomography (CT) scans. USG is used to accurately determine the location of lesions for aspiration. A CT scan of the abdomen is the most dependable imaging method for diagnosing splenic TB, which can accurately pinpoint the lesion’s location. On a CT scan, the splenic abscess typically appears as a low-density mass with peripheral enhancement upon the infusion of intravenous contrast [1,8]. The presence of acid-fast bacilli can be identified using techniques such as the ZN stain, polymerase chain reaction methods, and culture [4].

In the majority of splenic TB cases reported, the treatment approach has involved percutaneous aspiration or splenectomy followed by antitubercular therapy or just antitubercular therapy on its own. In all these instances, the vanishing of splenic hypodensity and a full response to the medication served as additional confirmation of the diagnosis of a TB splenic abscess [8]. Although splenectomy is seldom needed in the treatment of spleen TB, it might become necessary in patients with splenic abscess or spontaneous rupture of the spleen or if the treatment does not yield any response [5]. In our case, a splenectomy was carried out because of an impending rupture of a splenic abscess.

This case study underscores the occurrence of isolated splenic TB, encompassing its clinical manifestation, diagnosis, and treatment in an immunocompetent patient. It highlights the necessity for clinicians and healthcare workers to promptly and meticulously diagnose primary visceral TB.

## Conclusions

We have reported an uncommon instance of splenic TB in a man with a competent immune system who had no previous record of pulmonary TB. Primary TB splenic abscess is seldom diagnosed in patients with a competent immune system, and it is frequently misidentified due to the variable nature of its symptoms. Regardless of their HIV status, doctors should always keep TB in mind when caring for patients who present with splenic masses in regions where TB is prevalent. In cases of extrapulmonary TB where ZN stain results are usually negative due to their paucibacillary nature, highly sensitive methods such as cartridge-based nucleic acid amplification test (CBNAAT) ULTRA are beneficial for confirming the diagnosis.
